# Multi-nucleotide *de novo* Mutations in Humans

**DOI:** 10.1371/journal.pgen.1006315

**Published:** 2016-11-15

**Authors:** Søren Besenbacher, Patrick Sulem, Agnar Helgason, Hannes Helgason, Helgi Kristjansson, Aslaug Jonasdottir, Adalbjorg Jonasdottir, Olafur Th. Magnusson, Unnur Thorsteinsdottir, Gisli Masson, Augustine Kong, Daniel F. Gudbjartsson, Kari Stefansson

**Affiliations:** 1 Department of Molecular Medicine, Aarhus University, Denmark; 2 deCODE genetics/Amgen, Inc., Iceland; 3 Department of Anthropology, University of Iceland, Iceland; 4 School of Engineering and Natural Sciences, University of Iceland, Iceland; 5 Faculty of Medicine, University of Iceland, Iceland; Stanford University, UNITED STATES

## Abstract

Mutation of the DNA molecule is one of the most fundamental processes in biology. In this study, we use 283 parent-offspring trios to estimate the rate of mutation for both single nucleotide variants (SNVs) and short length variants (indels) in humans and examine the mutation process. We found 17812 SNVs, corresponding to a mutation rate of 1.29 × 10^−8^ per position per generation (PPPG) and 1282 indels corresponding to a rate of 9.29 × 10^−10^ PPPG. We estimate that around 3% of human *de novo* SNVs are part of a multi-nucleotide mutation (MNM), with 558 (3.1%) of mutations positioned less than 20kb from another mutation in the same individual (median distance of 525bp). The rate of *de novo* mutations is greater in late replicating regions (p = 8.29 × 10^−19^) and nearer recombination events (p = 0.0038) than elsewhere in the genome.

## Author Summary

In each generation new genetic variants are introduced by mutations. In this study we use whole genome sequence data from Icelandic families to directly observe such new mutations. Our estimate of the mutation rate implies that a newborn with 30-year-old parents will on average carry 75 new SNV mutations and 6 new short indel mutations. We observe that some of the found mutations occur much closer together than would be expected by chance. Our analysis shows that mutational hotspots cannot explain this clustering, instead the clustering mutations are likely created by a single mutational event. We observe a different composition for mutations that cluster very close together compared to more distant clustering mutations. This suggests that there is likely more than one type of underlying mutational mechanism creating the multi nucleotide mutation events. We furthermore observe a higher mutation rate near recombination events but find that this effect cannot explain the large number of clustering mutations that we observe.

## Introduction

Germline *de novo* mutations–i.e. mutations that occur during the formation of egg and sperm cells–are ultimately responsible for all heritable traits and evolutionary adaptations. Knowledge about the properties of these mutations is important for timing events in evolutionary history and understanding the causes of phenotypic diversity, such as disease. It is now possible to directly and comprehensively identify *de novo* mutations using whole genome sequence (WGS) data from nuclear families. Thus, we are now better equipped to answer questions such as: What is the mutation rate in humans? What affects the differences between the numbers and types of mutations within and between individuals? And are all mutations the result of independent events?

Several recent studies have estimated the mutation rate using WGS data from nuclear families [1–5]. However, calculating a rate estimate is not a trivial task and some uncertainty about the actual rate remains [6]. Comparisons between individuals show that paternal age explains most of the diversity in the number of *de novo* single nucleotide variants (SNVs), but it is less clear whether there are contributions from other factors such as maternal age or environmental exposure. Because *de novo* indels are somewhat rarer than *de novo* SNVs, and harder to detect, most studies have excluded them and less is therefore known about their mutation rate and the impact of factors such as parental age.

The germline mutation rate varies across the human genome at both fine and broad scales [6]. The most important factors affecting variation in the fine scale mutation rate are sequence context and methylation. At a broader scale, replication timing has been shown to be influential with significantly more mutations occurring in late-replicating regions [7]. The rate of recombination is positively correlated with genetic diversity in the human genome, but there has been much debate over whether this is due to increased efficiency of selection, confounding factors or a mutagenic effect of recombination [8–10].

It is often assumed that all mutations are the result of independents event even though several lines of evidence call this assumption into question [6]. Both family studies of germline mutations [3,11] and population data [12] have shown that mutations close to each other occur much more frequently than would be expected by chance, presumably due to single events that give rise to multiple nucleotide mutations (MNMs). In this paper we try to characterize such MNM events and estimate how common they are, which is important in order to understand their impact on human evolution and health. In addition we examine the potential mechanisms that could be responsible for different kinds of MNM events.

## Results

### The rate of de novo mutations

As a part of a large sequencing project in Iceland [13], we have obtained whole genome sequences (WGS) of 283 parent-offspring trios. Variant calling was performed using GATK and conservative filtering criteria were applied to identify a high-confidence set of autosomal *de novo* mutations (see methods). This resulted in a set of 17812 SNVs (avg. of 63 per individual), corresponding to a mutation rate of 1.29 × 10^−8^ per position per generation (PPPG), with a 95% confidence interval (c.i.) of 1.27 × 10^−8^ to 1.30 × 10^−8^. We also identified 929 short deletions and 353 short insertions (less than 35bp), corresponding to a combined indel mutation rate of 9.29 × 10^−10^ PPPG (c.i.: 8.79 × 10^−10^–9.82 × 10^−10^). This indel mutation rate falls between two recent estimates based on whole genome sequencing of trios [1,14]. The ratio between the *de novo* SNV rate and indel rate (13.78) is the same as that observed between the overall number of segregating SNVs and short indels in the Icelandic population (13.68)[13]. The denominators of the rate estimates were calculated separately for each individual and independently for SNVs and indels using a probabilistic method (see methods). Using this probabilistic method, we estimate that we can correctly identify 91.3% of the autosomal *de novo* SNVs and 90.9% of short *de novo* indels.

**Fig 1:**
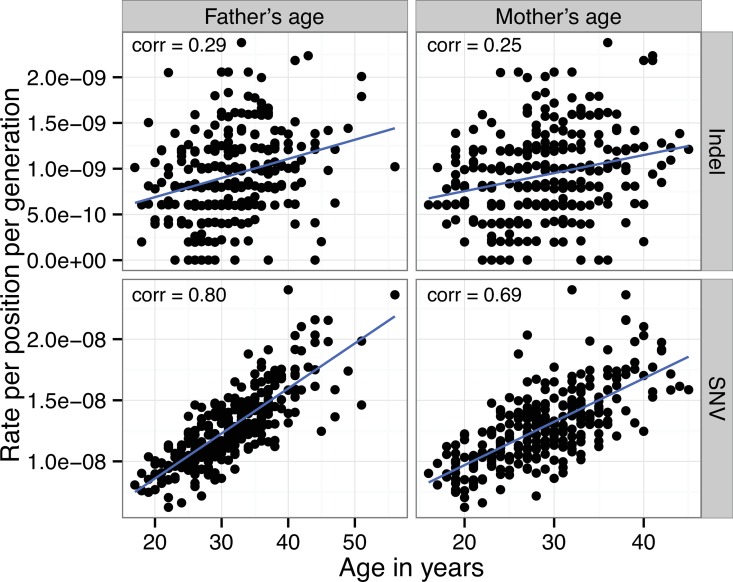
The correlation between the estimated mutation rate for each child and the age of the parents.

For both SNVs and indels, the rate of *de novo* events per offspring is positively correlated with the age of parents (see Fig 1). The ages of the parents are, however, highly correlated (r^2^ = 0.65). A multiple regression analysis shows that while the correlation with the mutation rate is primarily driven by father's age at conception (amounting to roughly 1.7 autosomal mutations for each additional year, p = 8.3 × 10^−26^), there is also a borderline effect of mother's age (~0.34 mutations per year, p = 0.041). Similarly, indels are primarily driven by father's age (~0.1 per year, 6.1 × 10^−3^), with a non-significant effect of mother's age (~0.01 per year, p = 0.75). The average age of the fathers and mothers is 31.6 and 28.9 respectively. The finding of a maternal age effect for SNV mutations is consistent with a recent study that found a maternal age effect of 0.35 mutations per year, but our estimate of the paternal age effect is significantly higher than what was reported in that study (0.64 additional mutations per year)[15].

As the number of mutations per generation is strongly affected by the length of the generation interval, it is more informative to report rates in the scale of years. We estimate the SNV mutation rate to be 4.25 × 10^−10^ (95% c.i.: 4.18 × 10^−10^–4.31 × 10^−10^) PPPY. This is slightly higher than previous estimates based on parent-offspring trios, but is nonetheless approximately a factor of 2 lower than the rate typically used to estimate speciation times between humans and closely related species [16]. Table 1 shows mutation rates per year for different types of mutations.

**Table 1:** The rate of different types of mutations.

Mutation Type	Sequence context	Transition vs. Transversion	Number of mutations	Mutation rate PPPY x 10^10^ (95% c.i.)	
SNV	CpG	Transition	2984	39.81	(38.40–41.26)
		Transversion	281	3.75	(3.34–4.21)
		All	3265	43.55	(42.09–45.07)
	nonCpG Strong (C or G)	Transition	4264	2.64	(2.56–2.72)
		Transversion	3019	1.87	(1.80–1.93)
		All	7283	4.50	(4.40–4.61)
	Weak (A or T)	Transition	4758	1.90	(1.85–1.96)
		Transversion	2506	1.00	(0.96–1.04)
		All	7264	2.91	(2.84–2.97)
Insertion	All		353	0.08	(0.08–0.09)
Deletion	All		929	0.22	(0.21–0.24)

The rates per position per year (PPPY) for different types of mutations. G and C base pairs are referred to as strong because they are bound by three hydrogen bonds while weak (A and T) base pairs are bound by two hydrogen bonds.

The estimated indel rate is 3.07 × 10^−11^ PPPY (95% c.i.: 2.91 × 10^−11^–3.25 × 10^−11^). The observed deletion to insertion ratio of 2.6 is consistent with a ratio of 2.8 observed for exonic *de novo* indels [17], but it is significantly higher than a ratio of ~2 estimated by using related species to determine the ancestral states of segregating human indels [18]. The observed distribution of indel lengths shows that the two most common types of *de novo* indels are 1 bp deletions (33.8%) and 1 bp insertions (19.7%) (see S1 Fig). The largest deletion that we observe is 35 bp long, while the largest insertion is only 8 bp long. The absence of larger insertions is probably because those variants are difficult to call using short read and therefore the true deletion to insertion ratio is likely to be smaller than our estimate.

### Multi-nucleotide mutation events

**Fig 2:**
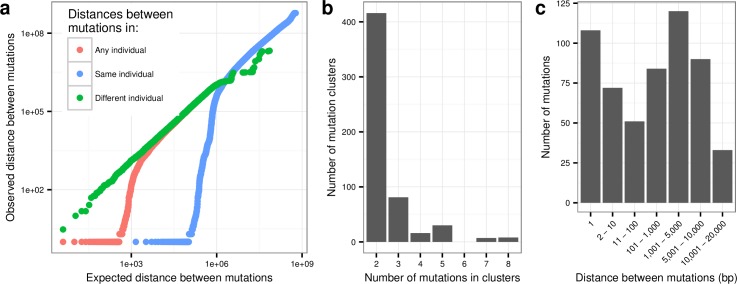
Clustering mutations. (a) The red line shows a QQ-plot of the observed distances between all pairs of mutations (both within and between individuals) compared to the expected distances assuming independence. The green line shows a QQ plot based only on distances between mutations that occurred in the same individual. The blue line shows a QQ plot based only on distances between mutations that occurred in different individuals. (b) A histogram of the number of mutations per cluster. (c) Histogram showing the distribution of distances to the nearest mutation in the same individual.

Mutations are typically assumed to occur uniquely and independently in the genome, even though several lines of evidence call these assumptions into question [6]. Testing for the random distribution of distances between neighboring *de novo* SNVs in our data, we see a significant overrepresentation of mutations close to each other. Fig 2A shows the distribution of observed distances compared to the null expectation that mutations occur independently. These results show that the clustering of mutations occurs solely within individuals and that the distances between mutations from different individuals are consistent with the null expectation. Thus, there is no sign of mutational sites shared by many individuals.

Clustering of mutations within individuals has been reported in previous studies and can be explained by MNM events [3,19–21]. One study estimated that 1.9% of all mutations are part of a MNM event with positions separated by less than 20bp [22]. Recent studies of *de novo* mutations found enrichment of mutations that are several kb apart [3,21] and it is possible that these more distant clustered mutations are also the result of a single mutational event. We find that 2.4% (435 of 17812) of *de novo* SNVs are accompanied by another mutation in the same individual less than 5kb away. Assuming that mutations are independent we would expect 0.03% (95% c.i.: 0.00%–0.07%) of mutations within this distance of each other. Using a less conservative criterion, we find that 3.1% (558 of 17812) of the *de novo* SNVs are accompanied by another mutation less than 20kb away compared to an expectation of 0.11% (95% c.i.: 0.03%–0.20%). We therefore conclude that around 3% of SNVs are likely the result of MNM events. This estimate is significantly larger (p = 3.5×10^−19^) than what was reported in a recent article [11] where 1.46% (161 of 11020) of mutations clustered within 20kb. This discrepancy cannot be fully explained by the fact that our study has higher sequence coverage and that our mutation calls thus have higher specificity. If we subsample our mutations so that we have the same average number of mutations per individual as in [11] (44.1 compared to our 62.9) we still observe that 2.4% (95% c.i.: 2.1%–2.6%) of mutations cluster within 20kb.

The 558 SNV mutations can be grouped into 247 MNM clusters, most of them with just two mutations, but with the largest cluster containing 8 mutations (see Fig 2C). The majority (315 of 558) of clustered mutations are less than 2kb from another mutation (median distance = 525 bp) and 17% (108) are immediately adjoining. Considering these 108 adjoining mutations as 54 tandem mutation events we estimate that the tandem mutation rate is 0.30% (95% c.i.: 0.23%–0.40%) of the single nucleotide mutation rate. This estimate is not far from the estimate of 0.4% that was recently calculated in a meta-analysis of 7 different studies that had estimated the tandem mutation rate [23].

We validated a subset of the 558 clustered mutations using Sanger sequencing. For 11 of the 57 pairs of clustered mutations that we tried to validate, sequencing failed in at least one trio member for at least one of the variants. The remaining 46 pairs of variants were all validated as genuine *de novo* variants that were present in the child, but not in the parents, yielding an estimated false discovery rate of 0.0% (95% c.i.: 0.0%–4.0%). The pairs were selected to validate both clusters spanning few bases and those spanning several kilobases, such that 19 were separated by <100bp, 19 between 100–2000 bp and the remaining 19 pairs between 2kb and 20kb.

We expect all the mutations in a MNM cluster to originate in the same parent and the alternative alleles should thus be found on the same chromosome. This can be tested when two mutations are close enough to be found on the same read or on paired-end reads from the same template molecule. We observed 159 pairs of positions with mutations (121 clusters) that were covered by single or paired-end reads. For 158 of these, the alternative alleles were found on the same template molecule (i.e. chromosome). For one pair of mutations, the information was contradictory with 4 reads supporting one phasing and 11 reads supporting a different phasing. The fact that the clustering mutations occur on the same chromosome supports our conclusion that they are created by a single mutational event.

If we consider both SNVs and indels together, 656 mutations (in 286 MNM clusters) are accompanied by another mutation in the same individual less than 20kb away. We observe 52 clusters that contain indels and 10 of these clusters consist solely of indels and the remaining 42 contain both SNVs and indels.

### Mutational mechanisms

**Fig 3:**
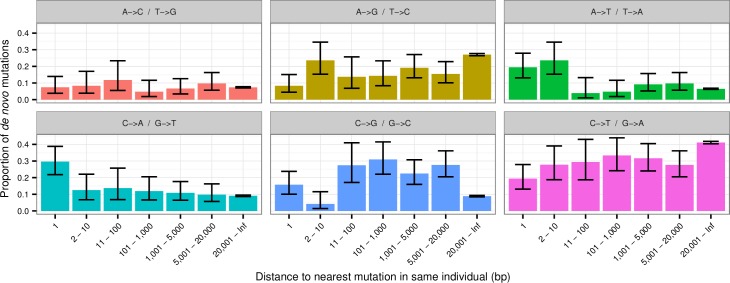
Distribution of mutation types. The relative distribution of different types of mutations stratified by the distance to nearest mutation in the same individual. The error bars are 95% confidence intervals.

We next examined more closely the mutations that cluster within individuals to gain insight into the mutational mechanisms that cause MNM events. Fig 3 shows the relative rates of all six possible types of SNV mutations, ignoring strand differences (i.e. C→T is the same as G→A). The results show that the frequencies of mutation types in clusters vary as a function of the distance between the mutations. Mutations less than 10bp from each other show a significant overrepresentation of A→T and the tandem mutations also show a significant overrepresentation of C→A mutations. Such a pattern is not observed in the 10bp-20kb categories, which instead show an overrepresentation of C→G mutations. The mutational spectrums for each of the different groups of clustering mutations shown in Fig 3 are all significantly different compared to the non-clustering mutations (the right most bin in Fig 3)(all comparisons have a p-value less than 3 × 10^−3^). Furthermore the tandem mutations are significantly different from all the other groups of clustering mutations and so is the group of mutations that cluster 2 to 10bp apart. These results suggest that different mutational mechanisms may underlie the tandem mutations, the mutations that are 2 to 10bp apart and the more distal clustered mutations.

#### Possible mechanisms creating tandem mutations

A recent paper described the use of population data to infer past MNM events in humans and reported a skewed distribution of ancestral and derived sites in tandem mutations [24]. They reported that GC→AA and GA→TT and their reverse complements were overrepresented and accounted for 27% of the tandem mutations. In our data, these two types of mutations make up 20% (11/54) of the tandem mutation pairs, which is much more than the 2.2% that would be expected if the mutations were independent (p = 2.3× 10^−8^). These two types of mutations have previously been shown to dominate the spectrum of mutations that are introduced by the error-prone polymerase ζ [25], suggesting that low-fidelity replication by polymerase ζ caused at least some of the tandem mutations that we observe. Experiments in yeast have also shown that polymerase ζ can produce complex mutations that include both tandem mutation and indels [25,26]. We observe that 13%(7/54) of the tandem mutations are accompanied with a *de novo* indel less than 10 bp away.

#### Possible mechanisms causing mutations occurring 10bp-20kb apart

**Fig 4:**
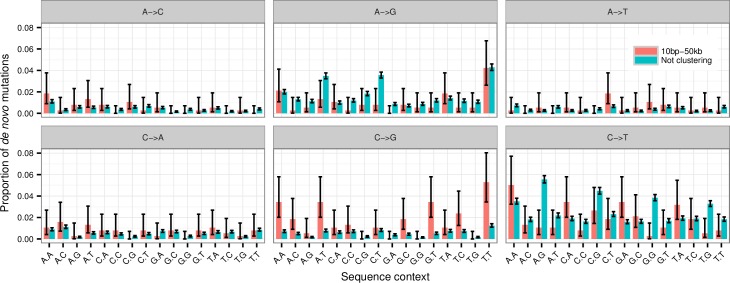
Distribution of mutation types with sequence context. The relative distribution of different types of mutation when the bases immediately 5’ and 3’ to the mutated base is included. Stratified based on whether the mutation clustered 10bp-20kb from another mutation or was not part of a cluster. The error bars are 95% confidence intervals.

The overrepresentation of C→G among clustering mutations was also noticed in [11], but the mechanism responsible for these remains unknown. The error prone polymerase REV1 is known to cause C→G mutations at abasic sites created by cytosine deamination and may thus be involved in the creation of these mutations [27,28]. The activity of the APOBEC cytosine deaminases is known to cause clusters of C→G and C→T mutations in several types of cancers by deaminating cytosines in stretches of single stranded DNA [29]. The deamination induced by APOBEC occurs primarily at TCW sites (the underlined base is the mutated base, and W means A or T). If we look at the mutations that cluster 10bp to 20kb apart we do not observe that a significantly larger fraction of the C→G mutations in this group occur at TCW sites (23.7%) compared to the non-clustering C→G mutations (22.2%) (p = 0.71). Nor do we observe a significantly increased fraction of clustering C→T mutations at TCW sites (p = 0.15). This means that we see no evidence of APOBEC activity being the cause of the cytosine deamination leading up to the cytosine mutations. Instead we observe a more general pattern with enrichment at cytosine positions that are not preceded by another cytosine or followed by a guanine base (i.e. DCH→G where D = A/G/T and H = A/C/T). 85.1% of the clustering C→G mutations match this DCH→G motif compared to only 68.3% of the non-clustering C→G mutations (p = 2.0×10^−4^). The results in Fig 4 also show that there are relatively fewer CpG transitions among the clustering mutations than the non-clustering. The percentage of C→T transitions that occur at CpG-sites is 41.8% (95% c.i. 40.6%–42.9%) for non-clustered mutations, but only 14.7% (95% c.i. 10.0%–21.2%) for clustered mutations. This is evidence that cytosine methylation is not responsible for the clustering mutations. Only two of the 168 clusters that contain mutations that are 10bp to 20kb apart also contain an indel within 20kb. So there is no sign that the mechanism(s) that creates the more distant clustering mutations is prone to create indel mutations.

#### Mutagenic effect of recombinations

Recombinations are known to sometimes give rise to large structural variants [30], but it is still uncertain whether they also produce *de novo* SNVs [9]. Using long-range phasing [31] to determine the phase and parental origin of alleles from SNPs typed on Illumina micro-array platforms, we were able to identify the number and location of the crossover events that occurred in the germ cells that gave rise to the offspring [32] in 266 of the 283 families. The resolution to which crossover events can be mapped depends on the distance between flanking heterozygote markers. In our micro-array genotype data, the average bin length that we can map a crossover to is 201kb. We used these data to test for an association between the locations of crossover events and MNM clusters within individuals. As we could not determine the parental origin of all the *de novo* mutations, we considered paternal and maternal events combined. Our results show that three of the mutations in MNM clusters are in the same bin as a crossover event. A permutation test shows that this is not significantly more than would be expected by chance. If we do not restrict the analysis to clustering mutations we observe that 83 of the *de novo* SNVs overlap with a recombination bin in one of the parents. This is 41% more than expected by chance (permutation test p-value = 0.0038). Thus, recombination appears to have a mutagenic effect, but this effect cannot explain the large number of clustering mutations that we observe.

#### Effect of replication timing

**Fig 5:**
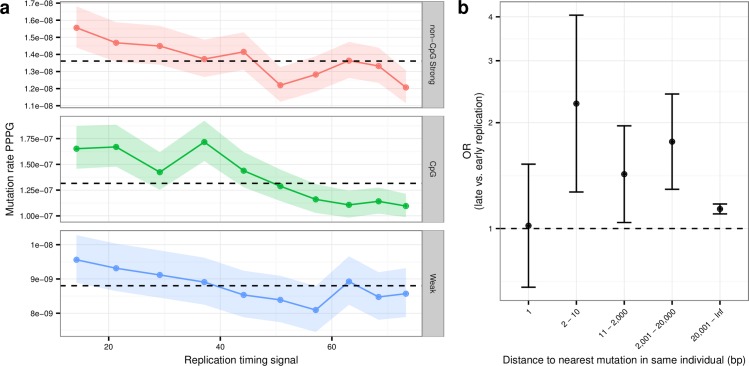
The effect of replication timing on the mutation rate. a) The effect of replication timing At CpG, non-CpG Strong (C or G) and Weak (A or T) sites. The y-values are mutation rates per position per year (PPPY). The x-values are wavelet-smoothed signal of replication timing calculated by the ENCODE project. Early replicating regions have high signal values and late replicating regions have low values. The bands around the points show the 95% confidence interval for each point. b) The effect of replication timing on clustering and non-clustering mutations. The y-axis shows a combined odds ratio for CpG, non-CpG-Strong and non-CpG-Weak sites calculated using the Cochran-Mantel-Haentzel method. An OR over 1 indicates that we observe most mutations in the latest replicating half of the genome. The error bars are 95% confidence intervals.

Late replicating regions of the human genome have previously been shown to have a higher SNP density [7] and to harbor more *de novo* mutations than the early replicating regions [11]. We used replication time data from the ENCODE project [33] to investigate the correlation with mutation rate in our data set. This analysis shows that the rate of mutation is significantly increased in late replicating regions both for mutations at CpG-sites and non-CpG sites (see Fig 5A) (combined p-value = 8.29 × 10^−19^). This association is stronger for the clustered mutations than the non-clustered mutations (Fig 5B). In particular, clustered mutations that are 1-10bp apart occur predominantly in late replicated regions of the genome. Contrary to a recent study we do not observe a different effect of replication timing in families with young fathers from that of families with old fathers [11] (See S2 Fig).

## Discussion

Recently there has been much interest in studying the human mutation rate using sequence data from nuclear families [1–5]. With WGS data from 283 Icelandic families, we have the resolution to study relatively rare *de novo* events such as indels and MNM events in more detail than article with smaller sample sizes. In addition to a large number of trios, the present study is also helped by the fact that sequence data are also available from a substantial fraction of the Icelandic population. These population data assist in assessing sequence quality and make it easier to weed out false positives.

Our study confirms previous reports that the rate of mutation is lower than the value of 1 × 10^−9^ PPPY that has often been used for dating of divergence times within and among humans and related species [16]. Some studies that use other methods have achieved slightly higher rates than the rate we report here [34,35], but studies based on sequencing of parent-offspring trios have consistently yielded estimates close to our estimate [1–4,15]. One implication of this is that some estimates of speciation times between humans and closely related species [36,37] are probably too low and should be adjusted upwards [16]. Our rate estimates were calculated using a probabilistic method that estimates the effective number of positions where a *de novo* mutation could be called [1]. This method adjusts the rate for false negatives caused by insufficient sequence coverage in some regions, but fails to account for mutations that cannot be called regardless of sequence coverage. We do not expect this to have a noteworthy effect on our estimate of the SNV rate, but it could be a problem for indels. Some indels are hard to call because reads containing them fail to be mapped correctly to the reference. It is thus likely that our estimate of the indel mutation rate is too low. Insertions are particularly hard to call based on short-read sequence data and this might explain why we observe a larger ratio of deletions to insertions than expected based on analysis of segregating indels.

We estimate that approximately 3% of human de novo mutations are part of MNM events. This calls into question the accuracy of population genetic models that assume all mutations to be independent events. Incorporating MNMs into methods that use the spatial distribution of mutations to estimate demographic parameters could, for example, make a substantial difference to the accuracy of such models [24]. The prevalence of MNM events also changes how we think about the evolution of functional alleles. If there is a fitness valley between the current amino-acid sequence of a protein and its optimal state, it is important to take the possibility of MNM events into account. Instead of having to take one step backwards and then two steps forward a MNM event makes it possible for the protein sequence to jump directly to the fitness peak in a single step [22]. Previous studies have in fact noticed protein sequence changes that appear to be caused by MNM events. The first report of this phenomenon was the observation that there is a higher rate than expected of switches between TCN and AGY codons at conserved serine residues and it is likely that these codon switches are explained by single synonymous MNM events rather than two independent non-synonymous mutations [19].

The rate of recombination is positively correlated with genetic diversity in the human genome, but it has so far been unknown whether these factors are causatively linked. Our study demonstrates that recombination has a mutagenic effect through increasing the rate of *de novo* SNV mutations. This result is in line with a recent study that reported an increase in genetic diversity around DNA double-strand break (DSB) hotspots [38]. We furthermore report a significantly greater mutation rate in late replicating regions, particularly in the case of MNM events.

Our investigation of MNM events suggests that there might be more than one type of underlying mutational mechanism. Our results corroborate earlier reports that the error-prone polymerase ζ is involved in the creation of tandem mutations. In the more distal clustering mutations that are more than 10bp apart we see observe excess of C→G mutations, which points to a mutational mechanism involving cytosine deamination followed by error-prone translesional DNA synthesis by REV1 and polymerase ζ.

## Methods

### Calling de novo variants

The criteria for calling a *de novo* mutation at any given position in trios, where the child is referred to as the proband, were as follows:

1. The proband has a genotype likelihood ratio lik (AR)/lik (RR) > 10^10^, where R denotes the reference allele and A the alternative allele.
2. For both parents the ratio lik (RR)/lik (AR) > 200.
3. None of the 2,636 sequenced individuals (excluding any descendants of proband) have a likelihood ratio: lik (AR)/lik (RR) or lik (AA)/lik (RR) > 10^4^. Note that this means that some recurrent mutations could have been filtered out, but it is expected that the number will be small.
4. There are at least 15 quality (> = 20) reads for the proband at the mutated site and at least eight reads in each of the parents.
5. The number of reads supporting an A allele call should make up at least 30% of the quality sequence reads in the proband.
6. The A allele should be observed on both strands in the proband.
7. Filter based on site quality metrics calculated by GATK: The ReadPosRankSum value (Z-score approximation of the Wilcoxon rank-sum test on the position of the alternative allele in the reads that support it compared to the position of the reference allele) should be between -6 and 6, the MQRankSum (Z-score approximation of the Wilcoxon rank-sum test of the mapping quality of the read with the alternative allele compared to the reads with reference allele) should be above 6 and the FS value (Fisher’s exact test on strand bias) should be below 20.

### Estimating rates

One possible source of error when analyzing WGS data is that all parts of the genome are not equally well covered by the sequencing. This means that there are parts of a genome where it is hard to correctly call a *de novo* mutation even if it is there. This needs to be taken into account when the denominator in mutation rate estimates is calculated. Instead of making a hard cutoff between callable and non-callable positions in the genomes we use a probabilistic approach to estimate the number of sites where a *de novo* mutation could be called [1]. The probability of calling site *x* as a *de novo* mutation, given that it is a true de novo mutation in family *f*, we name the *callability* and we denote it by $C_{f}^{de\;novo}\;(x)$. The callability is estimated independently for each family conditional on the depth of the three family members at the site using the method described in [1]. The number of callable sites in a given family is then the sum of the callability of all sites in that family. If *n*_*f*_ denotes the number of de novo mutations found in family *f*, the estimated rate per generation is:

$$rate_{PPPG}=\frac{\sum_{f\in \text{families}}n_{f}}{2\;\sum_{f\in \text{families}}\sum_{x\in \text{sites}}C_{f}^{\text{de novo}}\left(x\right)}$$

And the estimate of the rate per year is:

$${rate}_{PPPY}=\frac{\sum_{f\in \text{families}}n_{f}}{\sum_{f\in \text{families}}\left((p_{f}+m_{f})\sum_{x\in sites}C_{f}^{de\;novo}(x)\right)}$$

Where *p*_*f*_ and *m*_*f*_ are the paternal and maternal generation intervals in family *f*.

The callability is estimated separately for SNVs and indels, because indels are generally harder to call than SNVs. The average number of callable sites in a family is 2.45 billion for SNVs and and 2.43 billion for indels. The distribution of the number of callable sites for each family is shown in S3 Fig. When calculating the percentage of *de novo* mutations that we expect to identify, we use the number of non-N bases in hg18 (2.68 billiion) and thus ignore the telomeres and centromeres.

Confidence intervals for the rate estimates (and for proportions of mutations) were calculated using the Wilson method [39].

### Testing the distribution of distances between mutations

To calculate how many mutations are expected to be less than 20kb apart in the same individual, assuming independence, we randomly permuted the family identifier of the mutations 500 times. For each permutation we then counted the number of mutations that was less than 20kb from a mutation in the same individual and found the median of that number over the 500 permutations.

To calculate the distribution of distances between mutations assuming an independent distribution of mutations (Fig 2A), we simulated 500 random mutation sets where each individual had the same number of mutations at “CpG”, “non-CpG Strong (C or G)” and “Weak (A or T)” sites as in the original data set. For each of these three types of sites, the positions of mutations were drawn so that the probability that a certain position was mutated was proportional to the mean estimated callability of that position. To ensure that we always have the same number of distances in each simulation, we considered the genome as a one long string of concatenated chromosomes. To calculate the three different kinds of distances used in Fig 2A for a given data set we first sorted the mutations. To find the distance to the nearest mutation in any individual (red line) we then listed the distance between each mutation and the next mutation in the sorted list. To find the distance to the nearest mutation in the same individual (blue line) we for each mutation observed the distance to the next mutation in the list that occured in the same individual. To find the distance to the nearest mutation in a different individual (green line) we observed the distance to the next mutation in the list that was not in the same individual. To produce the values for the x-axis in Fig 2A, we then sorted the distances (for each of the three kinds of distances) for each of the simulated data sets and took the median over the 500 simulated data sets for each rank.

### Testing the effect of replication time on the mutation rate

We downloaded bigwig files of wavelet-smoothed signal of replication timing for five different individuals from the ENCODE project [33,40] (see supplement for list of files) and for each position in the genome we calculated the average value. To avoid spurious correlations based on differences in sequence context, the analysis was stratified into three context groups “CpG”, “non-CpG Strong (C or G)”, “Weak (A or T)”. For each group, we counted the numbers of mutated sites and calculated the sum of the callability for non-mutated sites for each possible replication time value (disregarding sites that did not have replication time data). A p-value was calculated using a logistic regression model with the context group as a covariate. To produce Fig 5A, showing the effect of replication timing on the mutation rate, the genome was split into deciles based on the replication time and the average replication time value was calculated for each of the ten bins. To produce Fig 5B, that shows the effect of replication timing on clustering mutations, we found the median value and split the data into late replicating and early replicating halves. We then used the Cochran-Mantel-Haentzel method to calculate the combined ORs across the three context groups. The same method was used to produce S2 Fig.

### Read-backed phasing of mutations

To investigate whether clustering mutations originated on the same chromosome we used pysam to find all read-pairs that spanned more than one clustering mutation. If at least 90% of the reads spanning a pair of mutations supported a particular phasing we considered that to be the true phasing of the pair.

### Comparing mutation spectra and mutation motifs

We used a Fisher’s exact test to compare the distributions of different mutation types shown in Fig 3. A Fisher’s exact test was also used when testing for enrichment of a specific kind of mutation motif.

### Testing the effect of recombination on the mutation rate

The recombination analysis was restricted to 266 (out of the 283) families where recombinations had been successfully mapped in both parents. For each family, the number of mutations falling within a recombination bin was counted. Then the family identifier of the recombination lists was permuted, so that the positions of the *de novo* mutations in one family could be compared to the recombination positions from another family. Permutations were performed 5000 times and a p-value was calculated by counting how many times the number of mutations falling within a recombination bin was higher than in the observed data. The expected number of mutations falling within a recombination bin was calculated as the median count in the 5000 permutations.

## Supporting Information

S1 Fig Length distribution of indels. (EPS)

S2 Fig Effect of replication timing vs. father’s age. (TIFF)

S3 Fig Distribution of effective number of callable sites. (EPS)

S1 Table The found *de novo* mutations. (XLSX)

S2 Table Clusters of *de novo* mutations. (XLSX)
