# Supplementary figures and images for: Multi-nucleotide de novo Mutations in Humans
Source: PLoS Genet. 2016 Nov 15;12(11):e1006315. doi: 10.1371/journal.pgen.1006315 (PMC5147774; doi:10.1371/journal.pgen.1006315)

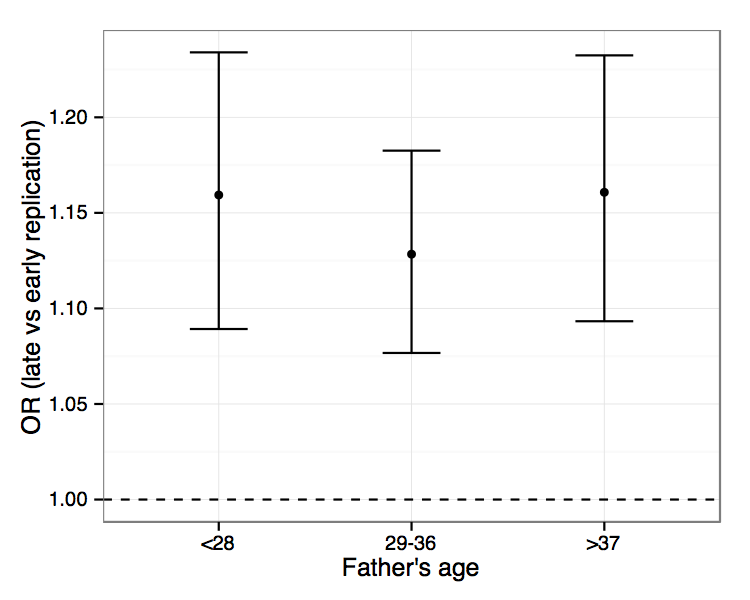

Supplement: S2 Fig — (TIFF) [file pgen.1006315.s002.tiff]
